# Antibacterial Mechanisms of Polymyxin and Bacterial Resistance

**DOI:** 10.1155/2015/679109

**Published:** 2015-01-15

**Authors:** Zhiliang Yu, Wangrong Qin, Jianxun Lin, Shisong Fang, Juanping Qiu

**Affiliations:** ^1^College of Biological and Environmental Engineering, Zhejiang University of Technology, Hangzhou 310014, China; ^2^Department of Electrical Engineering, Columbia University, New York City, NY 10027, USA; ^3^Shenzhen Center for Disease Control and Prevention, Shenzhen 518055, China

## Abstract

Multidrug resistance in pathogens is an increasingly significant threat for human health. Indeed, some strains are resistant to almost all currently available antibiotics, leaving very limited choices for antimicrobial clinical therapy. In many such cases, polymyxins are the last option available, although their use increases the risk of developing resistant strains. This review mainly aims to discuss advances in unraveling the mechanisms of antibacterial activity of polymyxins and bacterial tolerance together with the description of polymyxin structure, synthesis, and structural modification. These are expected to help researchers not only develop a series of new polymyxin derivatives necessary for future medical care, but also optimize the clinical use of polymyxins with minimal resistance development.

## 1. Introduction

An enormous and growing threat that some bacteria are becoming resistant to almost all available antibiotics is proposed to the world [1]. So far, there is no breakthrough in developing new drugs to kill multidrug-resistance (MDR) microorganisms, and the use of *β*-lactam, quinolone, or aminoglycoside is ineffective. The class of polymyxin antibiotics is increasingly considered as the final option of antibiotic therapy for MDR bacteria that are resistant to almost all other currently available antibiotics [2, 3]. Polymyxins consist of polymyxins A~E, of which polymyxin B and polymyxin E (colistin) are currently used as clinical medicines. In general, they have a narrow antibacterial spectrum mainly against the Gram-negatives [4].

Polymyxin is an old class of nonribosomal cyclic lipopeptide antibiotics originally discovered in 1947 [5]. Since 1959, polymyxin E has been used for the treatment of Gram-negative bacterial infection. However, in the 1970s, clinical use of polymyxin E and polymyxin B was limited due to their serious nephrotoxicity and neurotoxicity after parenteral administration. Together with the emergence of less-toxic aminoglycosides and other antipseudomonal agents [6], its parenteral use was almost completely abandoned in the 1980s. The revival of polymyxin has been coming since the mid-1990s, due to the lack of novel antibiotics against prevalent MDR Gram-negative bacteria [7]. However, the concerns on their nephrotoxicity and neurotoxicity still remain. Accordingly, colistin methanesulfonate (CMS), a prodrug, is typically applied, from which the active compound is slowly released in the blood.

Bacteria are usually able to evolve different strategies to sense, respond, and adapt to bactericidal agents including polymyxin. Therefore, novel polymyxin derivatives with less toxicity and higher bactericidal activity are highly desirable. This communication mainly aims to summarize and discuss the current understanding of antibacterial mechanisms of polymyxin and the corresponding bacterial resistance. We hope that this will serve as an up-to-date reference for researchers to develop polymyxin analogues with better antibacterial activity and less adaptable bacterial tolerance.

## 2. Polymyxin Structure and Synthesis

### 2.1. Chemical Structure

**Figure 1:**
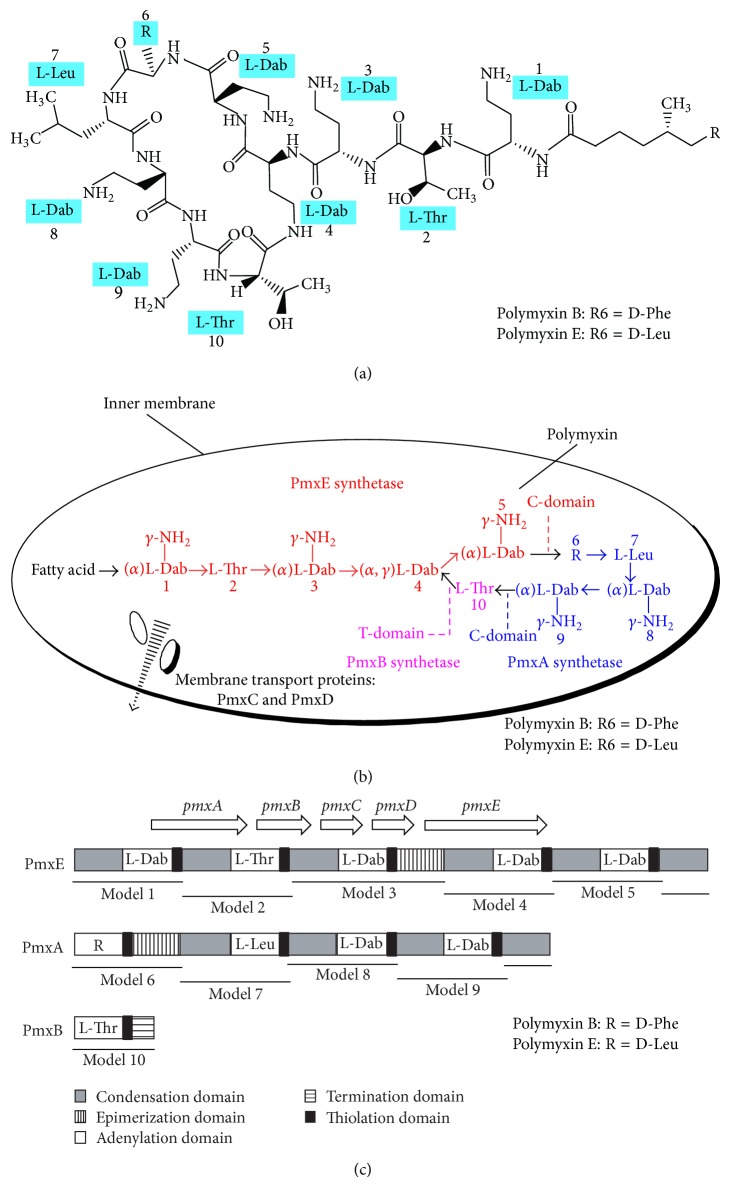
Representative polymyxin structure and its biosynthesis based on polymyxin B and polymyxin E [9]: (a) chemical structure; (b) polymyxin biosynthesis in* Paenibacillus polymyxa*; (c) gene cluster for polymyxin biosynthesis. Polymyxin is synthesized by three polymyxin synthetases, PmxA, PmxB, and PmxE, and transported by two membrane transport proteins, PmxC and PmxD. Fatty acid: 6-methyloctanoic acid or isooctanoic acid; Thr: threonine; Phe: phenylalanine; Leu: leucine; Dab: *α*,*γ*-diaminobutyric acid. The *α* and *γ* refer to the respective –NH_2_ involved in peptide linkage.

The structure of polymyxin is usually described as shown in Figure 1(a) due to the most thorough investigation on polymyxin B and polymyxin E. Its basic structure is a cyclic heptapeptide with a tripeptide side chain acylated by a fatty acid at amino terminus [8]. Polymyxin B and polymyxin E (Table 1) share almost identical primary sequence with major difference present at position 6 where D-Phe (D-phenylalanine) in polymyxin B is replaced by D-Leu (D-leucine) in polymyxin E [9]. The intramolecular cyclic heptapeptide loop is linked between amino group of side chain on diaminobutyric acid (Dab) residue at position 4 and carboxyl group of C-terminal L-Thr (L-threonine) residue at position 10. Therefore, its decapeptide sequence includes three parts, namely, a heptapeptide loop, a tripeptide side chain, and a fatty acid chain [10]. Polymyxin also bears other remarkable structural features, including cationic (L-*α*-*γ*-Dab) residues, making it polycationic at pH 7.4, and two hydrophobic domains (*N*-terminal fatty acyl chain and D-Phe^6^-L-Leu^7^ segment on polymyxin B or D-Leu^6^-L-Leu^7^ segment on polymyxin E). The mixture of lipophilic and hydrophilic groups makes it amphipathic [9, 10]. In addition, three-dimensional NMR analysis has revealed that polymyxin molecule is folded to form two distinct faces for polar and hydrophobic domains, thereby conferring structural amphipathicity that is essential for its antibacterial activity [9].

### 2.2. Polymyxin Biosynthesis

Different from ribosomal peptides that are synthesized by translation of mRNA, polymyxin is produced by nonribosomal peptide synthetase system (NRPS), a multienzyme complex with modular structures [11]. The typical module of NRPS mainly consists of three core domains: adenylation (A) domain, thiolation (T) domain (phosphopantetheine attachment site or peptidyl carrier protein), and condensation (C) domain. The A-domain plays a role in specific recognition and activation of amino acid or hydroxy acid through the formation of an aminoacyl adenylate. Then, the activated amino acid will be covalently bonded to 4′-phosphopantetheinyl (4′PPant) cofactor on T-domain via thioester formation. The T-domain mainly functions as transportation of substrate and elongation of intermediate to catalytic centers. Subsequently, the C-domain will catalyze the elongation of peptidyl chain by attaching thioesterified amino acid on phosphopantetheinyl arm at the upstream of T-domain to amino acid at the downstream of T-domain [12]. It is worth noting that NRPS can also include additional modules, such as epimerization and termination domains.

The modules and domains can orderly get together to form gene cluster. The biosynthetic gene cluster of polymyxin is called* pmx *cluster, including five open reading frames, namely,* pmxA*,* pmxB*,* pmxC*,* pmxD*, and* pmxE* (Figures 1(b) and 1(c)). Accordingly, they encode three polymyxin synthetases, PmxA, PmxB, and PmxE, and two membrane transport proteins, PmxC and PmxD [13]. PmxA comprises four modules whose amino acid substrates are Leu on polymyxin E or Phe on polymyxin B, Thr, Dab and Dab, and a C-domain. PmxB, responsible for the termination of polymyxin synthesis, composes only one module with Thr as its amino acid substrate. PmxE has five modules whose amino acid substrates are Dab, Thr, Dab, Dab, and Dab, and a C-domain. Based on the polymyxin structure, the order of modules for amino acid assembly during polymyxin synthesis should be PmxE-PmxA-PmxB [14], consistent with the order of ten amino acid groups on polymyxin molecule.

## 3. Polymyxin Derivatives 

As mentioned earlier, novel polymyxin derivatives with either higher antimicrobial activity or lower toxicity are highly promising. So far, researches on modification of polymyxin are mainly focused on the change of* N*-terminal fatty acyl chain length and hydrophobic domain of D-Phe^6^-L-Leu^7^ (polymyxin B) or D-Leu^6^-L-Leu^7^ (polymyxin E) and substitution of Dab side chains and amino acids [9].

The polymyxin toxicity is partly attributed to* N*-terminal fatty acyl segment [15]. The derivatives of polymyxin E with C9–C14 unbranched fatty acyl chains showed higher activity against polymyxin-resistant strains and Gram-positive bacteria with longer fatty acyl chain, whereas the derivatives with C10 and C12 fatty acyl chain were more effective against polymyxin-susceptible strains [16, 17]. The derivatives of polymyxin B with modified* N*-terminal fatty acyl chain have also been investigated to show that the analogue with intermediate length of* N*-terminal fatty acyl chain (octanoyl, C8) was optimal [18], while the ones with either longer (myristoyl, C14) [19] or shorter (acetyl, C2) [11, 20] chains displayed poorer antimicrobial activity. Moreover, the smaller acetyl nonapeptide analogues showed decreased antimicrobial activity against* Escherichia coli* and* Salmonella enterica*. Recently, it was revealed that, compared to polymyxin B with octanoyl (C8) fatty acyl chain [21], the analogues with* N*-terminal fatty acyl chains > C8 or 6-methyl moiety yielded decreased antimicrobial activity, due to the sterically hindered outer membrane (OM) insertion by fatty acyl moiety [22]. In addition, the substitution of* N*-terminus of polymyxin B with hydrophobic Fmoc group can significantly enhance antimicrobial activity and reduce toxicity [23].

The cationic Dab residue on polymyxin, particularly within the cyclic heptapeptide, plays a key role in polymyxin's antimicrobial activity through electrostatic interaction with phosphates of lipid A on bacterial member. The Dab on polymyxin has three important features, including cationic character of side chain groups, two-methylene group of Dab side chain, and specific order of Dab residues within the primary sequence that gives the proper spatial distribution of positive charge [9]. Various synthetic or semisynthetic modifications have been applied to Dab in order to increase antimicrobial activity or minimize potential toxicity [24, 25]. The* N* ^*γ*^-benzyl derivatives of polymyxin B and polymyxin E were synthesized by substituting Dab sides with lipophilic groups. Because of the reduced cationic character, the* N* ^*γ*^-benzyl derivatives appeared to have higher activity against Gram-positive* Staphylococcus aureus* and lower activity against Gram-negative* E. coli *[24]. The polymyxin B derivatives with positively charged or polar side chain on modified Dab showed better antimicrobial activity than polymyxin B and broadened the antibacterial spectrum [25]. It has been found thatthe Dabs within the heptapeptide ring on polymyxin B were more critical than the ones in linear tripeptide segment for antimicrobial activity [26]. As a kind of aminoglycoside, polymyxin carries 5 positive charges. Its nephrotoxicity is due to the highly cationic nature of molecule. Recently, it was reported that the polymyxin analogue with substitution of Dabs at positions 1 and 3 with Thr, Ser, or aminobutyryl group reduced its nephrotoxicity [27].

The hydrophobic domain of D-Phe^6^-L-Leu^7^ (polymyxin B) or D-Leu^6^-L-Leu^7^ (polymyxin E) can also affect its antibacterial activity through insertion with bacterial OM [26]. The hydrophobic domain of polymyxin B was evaluated by replacing D-Phe^6^ with D-Trp or D-Tyr and substituting L-Leu^7^ with L-Phe or L-Ala. The substitution of D-Phe^6^ and L-Leu^7^ with D-Tyr and L-Ala, respectively, significantly reduced LPS affinity and OM permeabilizing activity of polymyxin. The substitution of D-Phe^6^ with D-Trp, despite the similar affinity to LPS, displayed marginally reduced OM permeabilizing activity. The substitution of D-Phe^6^ with L-Phe resulted in an almost complete loss of OM permeabilizing activity [28]. It was reported that the replacement of D-Phe^6^-L-Leu^7^ segment with dipeptide mimics caused the loss of activity against* E. coli* [29].

Besides the above modifications, the size of cyclic peptide ring [30], the length of* N*-terminal linear tripeptide segment [31], and the generation of mimetic compounds [32] are also involved in polymyxin modification. A series of polymyxin B nonapeptide analogs with a cyclic peptide ring in size from 20 to 26 atoms were synthesized [30]. It was found that, among them, the one with native 23 atoms displayed the best OM permeabilizing activity and provided the most ideal structural configuration for potent antimicrobial activity. The analogues with a tripeptide linear tail of Met-Leu-Phe at* N*-terminus exhibited 8 to 10 times less toxicity than parent molecules [31]. The analogs of polymyxin B were designed to form amphipathic structure when they bind to LPS through tandemly repeated sequences of alternating cationic (Lys) and nonpolar (Val or Phe) residues [32]. It was found that the new analogs had strong antimicrobial effects but lacked hemolytic activity, highlighting the importance of peptide amphipathicity.

## 4. Antibacterial Mechanism of Polymyxins

### 4.1. Membrane Lysis Death Pathway

In Gram-negative bacteria, OM acts as a permeability barrier. The initial target of polymyxin is LPS of OM. Polymyxin can selectively bind to LPS, coincident with its narrow spectrum of antibacterial activity against Gram-negative bacteria [9]. LPS is composed of three domains: innermost lipid A, central core oligosaccharide region, and outermost O-antigen chain [33]. Among them, the most important domain is lipid A which serves as a hydrophobic anchor with tight packing of fatty acyl chains to stabilize overall OM structure. Some divalent cations such as Ca^2+^ and Mg^2+^ usually serve as a bridge between the adjacent LPS molecules to stabilize monolayer [34, 35].

**Figure 2:**
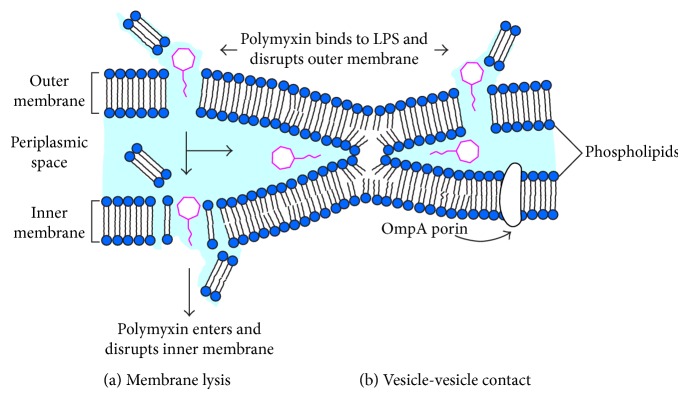
Antibacterial mechanisms of polymyxin: (a) classic mechanism of membrane lysis [9]; (b) alternative mechanism of vesicle-vesicle contact [39, 40]. The polymyxin is colored as magenta. LPS: lipopolysaccharide.

It is generally believed that the polymyxin kills bacteria through membrane lysis, as shown in Figure 2(a) (left). Firstly, the protonation of free *γ*-amines present on positively charged Dab residues provides a means of electrostatic attraction to negatively charged phosphate headgroups of lipid A, resulting in displacement of divalent cations (Ca^2+^ and Mg^2+^) [9, 10]. After this initial electrostatic interaction, the polymyxin molecule will insert its hydrophobic* N*-terminal fatty acyl chain and D-Phe^6^-L-Leu^7^ (polymyxin B) or D-Leu^6^-L-Leu^7^ (polymyxin E) segment into OM. This insertion will weaken the packing of adjacent lipid A, thus inducing the expansion of OM monolayer [10, 36]. Eventually, this facilitates the formation of destabilized areas through which polymyxin will cross OM [37, 38]. Finally, polymyxin will destroy the physical integrity of phospholipid bilayer of inner membrane (IM) through membrane thinning by straddling the interface of hydrophilic headgroups and fatty acyl chains [9], leading to IM lysis and cell death.

### 4.2. Vesicle-Vesicle Contact Pathway

An alternative mechanism, called vesicle-vesicle contact, has also been proposed [39, 40]. It is believed that polymyxin can mediate the contacts between periplasmic leaflets of IM and OM. The complex structure of OM consists of an inner phospholipid leaflet and an outer leaflet that predominantly contains LPS, proteins, and lipoproteins [10]. As shown in Figure 2(b) (right), polymyxin can bind to both anionic phospholipid vesicles, namely, inner phospholipid leaflets of OM and IM, and promote the exchange of phospholipids between vesicles. In brief, with the help of electrostatic interaction and two hydrophobic domains, the polymyxin molecule can enter into and cross OM. Then, polymyxin will induce the lipid exchange between leaflets of IM and OM, triggering the loss of specificity of phospholipid composition. This can potentially cause an osmotic imbalance, leading to cell lysis [39, 40]. It was reported that an analogue of polymyxin B with an intervening Dab residue in D-Phe^6^-L-Leu^7^ domain was much more effective in inducing lipid exchange through vesicle-vesicle contact and gave higher permeabilizing activity [41]. Another analogue of polymyxin B with substitution of D-Phe^6^ with D-Trp can bind to bacterial vesicles and induce the formation of vesicle-vesicle contact [42].

### 4.3. Hydroxyl Radical Death Pathway

**Figure 3:**
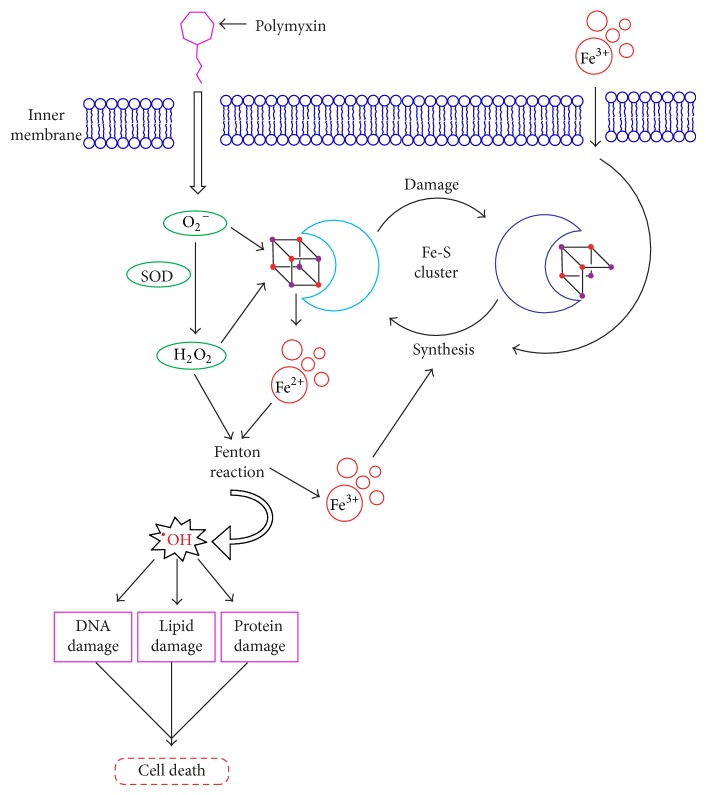
Hydroxyl radical death mechanism in bacteria induced by polymyxin [46]. The polymyxin molecule (magenta) comes across inner membrane (IM) and induces O_2_^−^ (superoxide) generation. Then, O_2_^−^ will be converted to H_2_O_2_ by SOD (superoxide dismutase). Both O_2_^−^ and H_2_O_2_ can oxidatively attack Fe-S (iron-sulfur) clusters (iron and sulfur are shown as red and purple points, resp.) and cause inactivation of Fe-S cluster (from light cyan to dark blue) and iron leaching. With the conversion of “free” ferrous iron (Fe^2+^) to ferric iron (Fe^3+^), H_2_O_2_, via Fenton reaction, will be rapidly converted to ^•^OH (hydroxyl radical) which readily damages DNA, lipids, and proteins.

A new report showed that polymyxin can possibly induce rapid cell death through the accumulation of hydroxyl radical (^•^OH) (Figure 3). This hypothesis is based on the oxidative stress due to polymyxin-induced formation of reactive oxygen species (ROS), including superoxide (O_2_^−^), hydrogen peroxide (H_2_O_2_), and ^•^OH in Gram-negative bacterial cells [43]. It has been hypothesized that O_2_^−^ will be induced when polymyxin molecules enter into and cross OM and IM [44, 45]. Then, O_2_^−^ will be converted to H_2_O_2_ by superoxide dismutases (SOD) present in cells. Subsequently, H_2_O_2_ will oxidize ferrous iron (Fe^2+^) to ferric iron (Fe^3+^), along with the formation of ^•^OH, which is called Fenton reaction [44, 45]. When the concentration of ^•^OH reaches an uncontrollable level, it will result in oxidative damage of DNA, lipids, and proteins and eventually cause cell death [44, 46]. In this process, the damage and resynthesis of Fe-S dependent proteins, especially Fe-S dependent dehydratase, such as dihydroxy-acid dehydratase (DHAD), are important. The exposed Fe-S cluster will be oxidized by O_2_^−^ to an unstable species with H_2_O_2_ formation and Fe^2+^ release. Similar to O_2_^−^, H_2_O_2_ can also destroy the Fe-S cluster, leading to the loss of Fe^3+^ and inactivation of Fe-S dependent protein [43]. After damage by either O_2_^−^ or H_2_O_2_, the inactive Fe-S cluster can be repaired by protein YggX (a member of the SoxRS regulon) and a di-iron protein YtfE in the presence of Fe^3+^ [43], whose uptake will be strongly triggered by ferric uptake regulator. It has been demonstrated that the ^•^OH production will increase in polymyxin B- or polymyxin E-treated* Acinetobacter baumannii*, leading to rapid cell death [47]. Moreover, the killing of* A. baumannii *by polymyxins was delayed in the presence of inhibitors that can both directly and indirectly block the ROS production.

## 5. Mechanisms of Bacterial Resistance to Polymyxins

### 5.1. PhoP-PhoQ Two-Component System

**Figure 4:**
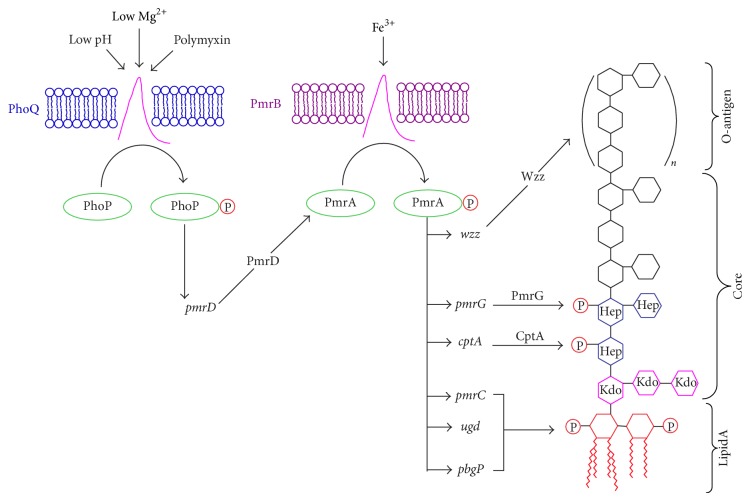
PhoP-PhoQ two-component system of bacterial resistance to polymyxin [57]. PhoP is phosphorylated by PhoP-PhoQ system under low Mg^2+^, low pH, and polymyxin, promoting* pmrD* gene to express PmrD protein. With the help of PmrD and in the presence of Fe^3+^, transcription of PmrA-activated gene is induced by PmrA-PmrB system. After phosphorylation, PmrA-P activates transcription of LPS modification loci (i.e., Wzz, PmrG, CptA, ugd, pbgP, and pmrC). The O-antigen synthesis is controlled by products of* wzz* gene. The PmrG and CptA proteins are responsible for the phosphorylation modification of heptose (I) and heptose (II) (blue segments), respectively. Lipid A (red part) can be phosphorylated with phosphoethanolamine (pEtN) encoded by PmrC or L-4-aminoarabinose (L-Ara4N) encoded by Ugd and PbgP. P: phosphorylated.

It is becoming increasingly clear that polymyxin resistance in Gram-negative bacteria involves the multitier upregulation of a number of regulatory systems [48, 49]. The OM usually serves as a permeability barrier to protect Gram-negative bacteria from various antibiotics and chemicals [34]. The critical step of bactericidal activity of polymyxin is the electrostatic interaction between positively charged Dab residues on polymyxin and negatively charged phosphate groups on lipid A of LPS [9]. The bacterial cell is able to reduce the initial electrostatic attraction by reducing net negative charge of OM via lipid A modification, thereby increasing resistance to polymyxin. The most common polymyxin-resistance mechanism inbacteriais attributed to the shielding of phosphates on lipid A with positively charged groups, such as phosphoethanolamine (pEtN) and L-4-aminoarabinose (L-Ara4N) [50–53], which is mediated by PhoP-PhoQ regulatory system encoded by* phoP* locus (Figure 4).

Activated by PhoP-PhoQ, the PmrA-PmrB encoded by* pmrCAB* operon is the major regulator to mediate the LPS modification in Gram-negative bacteria [54]. PmrA-dependent modification can occur on each of the three distinct LPS domains, namely, lipid A, core polysaccharide, and O-antigen chain. In the innermost lipid A, the interaction of either pEtN or L-Ara4N with lipid A will neutralize lipid A phosphates and confer resistance to polymyxin B [33, 55]. The* ugd* gene encoding UDP-glucose dehydrogenase and* pbg* gene encoding L-Ara4N transferase are both activated by PmrA. They are necessary for biosynthesis and incorporation of L-Ara4N [55]. On the other hand, an IM PmrC protein encoded by PmrA-activated* pmrC* gene is needed for pEtN incorporation into lipid A. In the central core polysaccharide region, the decoration of heptose (I) phosphate with pEtN can further increase the resistance to polymyxin B [56]. The PmrA-activated* cptA* gene encoding for pEtN phosphotransferase specific for the core is responsible for the modification of heptose (I) phosphate with pEtN. Moreover, the PmrA-activated PmrG protein which is normally introduced by RfaY protein is a phosphatase for removing the phosphate from heptose (II) phosphate [33]. In the outermost O-antigen chain, the increase of O-antigen length will result in the heightened resistance to polymyxin B, which can be boosted up by iron. The O-antigen synthesis of* S. enterica* is controlled by the products of* wzz*_*st*_ and* wzz*_*fepE*_ genes that are controlled by PmrA-PmrB regulatory system [57, 58]. The transcriptional induction of* wzz*_*st*_ and* wzz*_*fepE*_ is activated by PmrA through directly binding to their promoter, consequently increasing the amount of O-antigen in LPS and finally increasing resistance [57, 58].

The PhoP-PhoQ two-component system in* S. enterica* has been well characterized [54]. It acts as a master regulator of virulence and evasion of killing by polymyxin [59]. In response to sublethal concentrations of polymyxin, PhoQ, an IM sensor kinase, will phosphorylate the cytoplasmic regulator PhoP, leading to activation of PmrA-PmrB via PhoP-activated PmrD protein whose product affects the phosphorylation of PmrA [54, 60–63]. Under extracytoplasmic Fe^3+^ or Al^3+^ and acidic pH [64, 65], the sensor PmrB promotes phosphorylation of its cognate regulator PmrA, resulting in the transcription of PmrA-activated genes [66] and repression of PmrA-repressed genes [67]. Consequently, the PmrA-PmrB system activates the expression of PmrC or Ugd/PbgP, necessary for the covalent modification of phosphate groups on lipid A [68]. In addition, the PmrA-PmrB system will use PmrR to inhibit the activity of LpxT, a constitutively synthesized IM enzyme that generates diphosphorylated lipid A at 1-position (1-PP) [69]. All these PmrA-regulated modifications will decrease the overall negative charge of LPS, thereby avoiding the interaction with positively charged Dab residues of polymyxin. Upon the removal of stress from polymyxin, the phosphorylated PmrA (PmrA-P) in cells will be downregulated to appropriate level through three ways. Firstly, the PmrA-P protein can be positively downregulated through transcription of* pmrCAB* operon [70]; secondly, the PhoP-PhoQ two-component system can control the expression of* pmrD* gene to repress PmrA-P protein [71]; thirdly, as an intrinsic feedback mechanism, PmrB will dephosphorylate PmrA-P [67].

### 5.2. Species-Specific Resistance Mechanisms

Besides the LPS-binding pathway regulated by PhoP-PhoQ system, there are other unique and often species-specific mechanisms in polymyxin resistance. Multidrug efflux pumps play an important role of polymyxin resistance in Gram-negative and Gram-positive pathogens. The MexAB-OprM efflux pump in* Pseudomonas aeruginosa* has been proposed to confer tolerance towards polymyxin E, due to the increase of mexAB-oprM expression in* P. aeruginosa* upon polymyxin E exposure [72, 73]. The AcrAB efflux pump encoded by* acrAB* operon can give* Klebsiella pneumoniae* resistance to polymyxin B [74]. Moreover, the AcrAB efflux pump is also associated with polymyxin resistance in* E. coli* [75]. A multidrug efflux pump NorM in* Burkholderia vietnamien* has been shown to contribute to polymyxin resistance [76]. All these efflux pumps are thought to transport and pump out polymyxins present in cells.

In addition, polymyxin resistance is also thought to be associated with the expression of OM proteins in bacteria. It has been believed that the OM protein OprH, a membrane stabilization protein, can promote resistance to polymyxin B in* P. aeruginosa* [77]. The OM protein OmpA in* K. pneumoniae* can help to clearinfections, conferring resistance to antimicrobial peptides [78]. It has been found that the absence of OmpA decreases the expression of capsule polysaccharide, thereby increasing susceptibility to polymyxin B [79]. The capsule polysaccharide could increase resistance of* K. pneumoniae* to polymyxins [80]. Since the capsule polysaccharides are anionic whereas polymyxins are cationic, the capsule polysaccharides can bind to polymyxin to reduce the amount of peptides reaching bacterial surface. This will neutralize the bactericidal activity of polymyxin, at last enhancing electrostatic interaction between capsule polysaccharide and polymyxin [81].

Recently, It was found that the complete loss of LPS could lead to high-level polymyxin E resistance in* A. baumannii*, clearly indicating that the interaction of polymyxin E with LPS is critical for bactericidal action against* A. baumannii* [82, 83]. It is believed that the complete loss of LPS will decrease the target ability of polymyxin to cell, thereby causing high-level polymyxin resistance.

## 6. Future Perspective

The usefulness of polymyxin B and polymyxin E has been clearly demonstrated by optimizing their clinical use and developing their derivatives with less nephrotoxicity than earlier believed and they have been used as bactericidal agents for around 5 decades. Though polymyxins are mainly applied to killing Gram-negative pathogens, there are increasing reports showing their anti-Gram-positive bacteria activity. This needs to be further investigated for better understanding, because much higher concentration of polymyxin is needed against Gram-positive bacteria than the one against Gram-negative bacteria.

Different from traditional membrane lysis mechanism in bacteria, the ^•^OH accumulation is a newly proposed mechanism for polymyxin-induced cell death. However, the pathway to induce ^•^OH generation in cells exposed to polymyxin is still unclear. Since Fenton reaction is considered as the possible pathway for ^•^OH formation, it is very desirable to carry out detailed characterization on the key components such as SOD, H_2_O_2_, and Fe-S cluster in this reaction to fully understand this new mechanism.

Different mechanisms of polymyxin-resistance have been found in bacteria. Resistance to the current polymyxins could become a big global health challenge, because this means that virtually no antibiotics will be available for treatment of serious infections caused by polymyxin-resistant “superbugs.” Therefore, development of a next generation of polymyxin is urgently required. In order to achieve this goal, deeper understanding of the mechanisms of polymyxin antibacterial activity and bacterial resistance is the first and most crucial step.

## Figures and Tables

**Table 1:** The structural differences between polymyxin B and polymyxin E.

Polymyxin	Fatty acid^a^	R6^b^
Polymyxin B1	MOA	D-Phe
Polymyxin B2	IOA	D-Phe
Polymyxin E1 (colistin A)	MOA	D-Leu
Polymyxin E2 (colistin B)	IOA	D-Leu

^a^MOA: 6-methyloctanoic acid; IOA: isooctanoic acid; Phe: phenylalanine; Leu: leucine.

^b^R6 means amino acid residue at position 6 on polymyxin.
